# The use of accelerometry as a tool to measure disturbed nocturnal sleep in Parkinson’s disease

**DOI:** 10.1038/s41531-017-0038-9

**Published:** 2018-01-10

**Authors:** Sarah McGregor, Philip Churchward, Katarzyna Soja, Denise O’Driscoll, Michelle Braybrook, Hamid Khodakarami, Andrew Evans, Parisa Farzanehfar, Garun Hamilton, Malcolm Horne

**Affiliations:** 1 St Vincent’s Hospital Melbourne, Fitzroy, VIC 3065 Australia; 2 Global Kinetics Corporation (GKC), Melbourne, VIC 3000 Australia; 3 Florey Institute of Neuroscience and Mental Health, Parkville, VIC 3052 Australia; 4 Sahlgrenska Academy, Institute of Neuroscience and Physiology, Göteborg, Sweden; 5 Department of Respiratory and Sleep Medicine, Eastern Health, Box Hill, VIC 3128 Australia; 6 Eastern Health Clinical School, Monash University, Clayton, VIC 3800 Australia; 7 Royal Melbourne Hospital, Parkville, VIC 3050 Australia; 8 Department of Lung and Sleep, Monash Health, Clayton, VIC 3168 Australia; 9 School of Clinical Sciences, Monash University, Clayton, VIC 3800 Australia; 10 Department of Medicine, St Vincent’s Hospital, University of Melbourne, Parkville, VIC 3010 Australia

## Abstract

Sleep disturbances are common in Parkinson’s disease (PD). We used the Parkinson’s KinetiGraph (PKG), an objective movement recording system for PD to assess night time sleep in 155 people aged over 60 and without PD (controls), 72 people with PD (PwP) and 46 subjects undergoing a Polysomnogram (PSG: 36 with sleep disorder and 10 with normal sleep). The PKG system uses a wrist worn logger to capture acceleration and derive a bradykinesia score (BKS) every 2 min over 6 days. The BKS ranges from 0–160 with higher scores associated with lesser mobility. Previously we showed that BKS > 80 were associated with day time sleep and used this to produce scores for night time sleep: Efficiency (Percent time with BKS > 80), Fragmentation (Average duration of runs of BKS > 80) and Sleep Quality (BKS > 111 as a representation of atonia). There was a fair association with BKS score and sleep level as judged by PSG. Using these PKG scores, it was possible to distinguish between normal and abnormal PSG studies with good Selectivity (86%) and Sensitivity (80%). The PKG’s sleep scores were significantly different in PD and Controls and correlated with a subject’s self-assessment (PDSS 2) of the quality, wakefulness and restlessness. Using both the PDSS 2 and the PKG, it was apparent that sleep disturbances were apparent early in disease in many PD subjects and that subjects with poor night time sleep were more likely to have day time sleepiness. This system shows promise as a quantitative score for assessing sleep in Parkinson’s disease.

## Health technologies: Measuring sleep disturbances

A movement recording system reveals the occurrence of sleep disturbances in the early stages of Parkinson’s disease (PD). Malcolm Horne, a movement disorders expert at the University in Melbourne, and colleagues assessed night time sleep in 72 patients with PD using a wrist-worn device that captures movement patterns. The Parkinson’s KinetiGraph (PKG) system derives scores that are associated with sleep stages and correlate with patients’ self-assessment of sleep quality, wakefulness and restlessness. Significant differences between the PKG sleep scores of PD patients and age-matched healthy controls confirmed that night time sleep disturbances and day time sleepiness worsen as the disease progresses. A*bnormal PKG scores were found* in patients affected by the disease for only 3 years highlighting the extent to which sleep is disrupted in *early-stage PD*.

## Introduction

Disturbances of nocturnal sleep and daytime somnolence are common in people with Parkinson’s disease (PD).^1^ However much of the information about the extent to which sleep is disturbed in PD has been gleaned from self-reported questionnaires,^2^ and does not always concord with objective assessments.^3,4^ However questionnaires are frequently designed to understand why sleep is disturbed rather than to assess the duration and quality of sleep per se: for example, the Parkinsons disease Sleep Score 2^2^ (PDSS 2) has only 3/15 questions about sleep duration and quality, and the others relate to factors that cause sleep disruption. On the other hand polysomnography (PSG) studies and other forms of objective assessment are mainly directed at measuring duration and architecture of sleep. While PSG studies of people with PD (PwP) are mainly small or lack case controls,^3^ one large case controlled study^5^ did confirm that patients had shorter total sleep time, lower sleep efficiency and increased rapid eye movement (REM) sleep latency compared to controls; while nocturnal arousals, obstructive sleep apnea, periodic leg movements and objective abnormal sleepiness were not more frequent in patients than controls.^6^ Two important considerations could confound this finding of reduced sleep time and sleep efficiency in PD. First, PSG is usually for a single night and may suffer from the 'first night effect' of sleeping in the sleep lab, whereas it has been demonstrated that subsequent nights have ~30 mins more sleep duration with half of this in REM sleep.^7–10^ Second, age has a clear effect on sleep patterns^11^ with total sleep time reduced by about 10 min with every advancing decade, with a trend toward increasing light sleep and less slow wave or REM sleep.^11^ This is relevant because PwP are older than the usual subject undergoing a PSG study.

Actigraphy has been validated for estimating parameters of night-time sleep in populations of healthy adults^12,13^ but it performs less well in specific subpopulations such as those with PD^14^ or the elderly.^15^ Accelerometry can be used to quantify daytime sleep in PD.^16^ In night time sleep in PD, it performed well against clinical scales^17,18^ at a population level but was less successful as a tool for assessing all PSG defined parameters of sleep in a single individual with PD.^14^ This has led to the view that specific subpopulations may require specific calibration to assess sleep. Most studies using accelerometry to examine nocturnal sleep in PD have used standard devices calibrated for assessing sleep in the well subject or in the younger person with obstructive apnea. To our knowledge, none of these systems attempt to provide consideration of the extent to which atonia measured by attenuated acceleration signals can be used as a surrogate of sleep architecture. The Parkinson’s KinetiGraph is an accelerometry based system developed for measuring the movements of PD and provides a score of immobility consistent with sleep. Acceleration levels defined as 'immobile' in this system have been validated against ambulatory PSG for day time sleep^16^ and against clinical scales for nocturnal sleep.^18^ In this study, we have developed and calibrated scores of night time sleep using the PKG system in populations undergoing PSG, in subjects with PD and in a control population aged over 60.

## Results

### PKG sleep scores in Control subjects

**Fig. 1:**
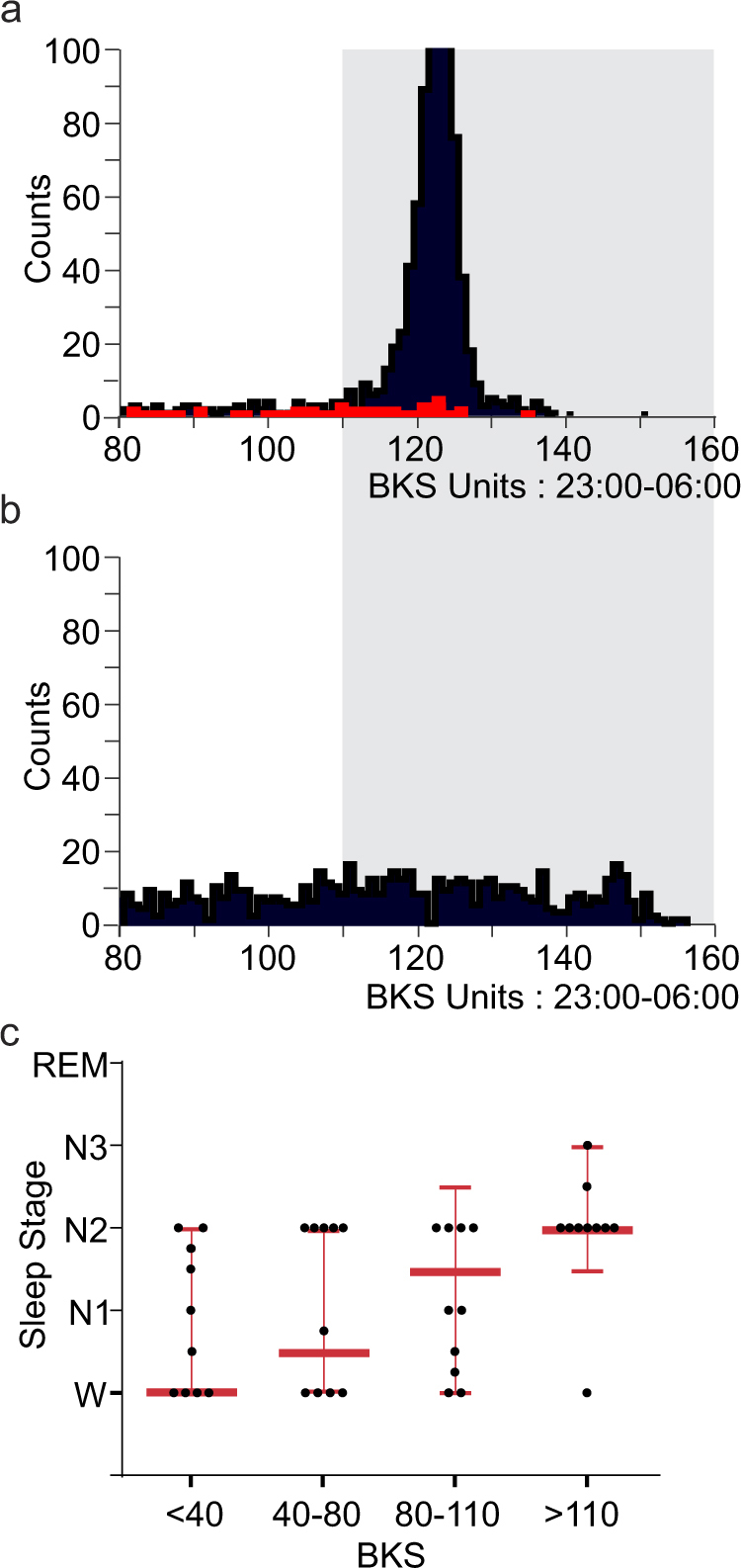
Immobility during sleep in a normal and a PD subject and a comparison with sleep stages. Figures 1a, b are frequency histograms of BKS (>80) obtained from 6 days of recording. The *X* axis is the value of the BKS unit and the *Y* axis the number of BKS units with that value. The grey shaded region shows BKS > 110. **a** These are two histograms obtained from a non-PD subject. The black bars show the frequency of BKS of different values from the night period (23:00–06:00) and the red bars are the BKS from the day period in the same individual. It shows a marked peak in BKS at approximately 120 in the night period which is not present in the day period. **b** A histogram from a PWP. The BKS > 80 are from the night period (23:00–06:00). Note that inactive BKS are reduced but the most obvious feature is the loss of the peak of BKS with scores >110. **c** This represents the findings of 10 individuals whose PSG was reported as normal. Each 2 min required for a BKS score is associated with four PSG scores of sleep state. The PSG sleep scores were given an ordinal value (awake = 0 and REM = 4) and the median 'PSG value' of the four associated with each BKS was estimated. For each individual, the median PSG value for all BKS in each of the four BKS categories was calculated (each dot represents an individual). The heavy horizontal red lines indicate the median of all 'PSG values' (i.e., each score from each individual pooled) of each BKS state (the lighter lines are the interquartile ranges). Thus, the median PSG value for BKS < 40 was 'awake'; for BKS 40–80 was between awake and N1; for BKS 80–110 was between N1 and N2; and for BKS > 110 was N2

In most actigraphy systems, periods of low acceleration are regarded as 'sleep' . Previously we showed that BKS > 80 in the day time, were associated with a high probability of sleep as measured by ambulatory PSG.^16^ Therefore, in this study, a BKS = 80 was used as the boundary between putative 'sleep' and 'wakefulness'. The distribution of BKS > 80 in daytime and night periods were compared (Figs. 1a, b). In the daytime, BKS were usually between 80–110, whereas in the night period they were clustered in an approximately bell-shaped distribution whose mode in normal subjects was usually above BKS = 120. In normal subjects, 75% of night period BKS ≥ 111, but BKS ≥ 111 were often attenuated or absent in PwP (Fig. 1b).

Because BKS > 111 are typically seen at night, the relationships between stages of sleep and BK scores were examined in 10 subjects whose sleep during a PSG was reported as normal. The PSG is scored on electroencephalography activity every 30 s as being awake or in a stage of sleep (N1, N2, N3 or REM). The contemporaneous 2 min BKS therefore overlapped four 30 s sleep scores. To compare the BKS with the Sleep Score, the four sleep stages and wakefulness were given an ordinal value (awake = 0 and REM = 4) and the median value of the four sleep stages that overlapped a single BKS was calculated (Table 1 and Fig. 1c).

**Table 1:** The PKG’s Bradykinesia scores in various PSG sleep states percent time of ALL BKS in each sleep stage, sorted according to BKS range

Percent time	BKS < 40	BKS 40–80	BKS 80–110	BKS > 110	Any BKS range
REM	1.7	0.9	1.8	8.1	12.6
S3	1.3	0.5	0.8	16.8	19.4
S2	4.1	2.2	2.9	25.4	34.7
S1	1.2	0.5	0.7	3.5	5.9
Awake	9.1	4	3.8	10.7	27.5
Percent time of each BKS range spent in each of the sleep states					
Sleep State	<40	40–80	80–110	>110	
REM	9.6	11.3	18.2	12.6	
S3	7.7	6.6	7.6	26	
S2	23.4	27.4	29.2	39.5	
S1	7	5.7	7.2	5.4	
Awake	52.3	49.1	37.9	16.5	

This showed that BKS < 80 were mostly associated with being Awake or in N1 (~60% of the time). Nevertheless ~25% of the time, BKS in this range was associated with N2 stage and with REM sleep in ~15%. Indeed, similar statements could be made for BKS < 111. The pattern was clearly different for BKS > 110, when 78% of BKS were associated with one of N2, N3 or REM sleep states. Furthermore, 90% of N3 stage and 60% of REM sleep occurred when BKS > 110. Nevertheless, people were awake ~16% of the time when the BKS > 110 (39% of all wakefulness occurred when BKS > 110). The overarching conclusions are that when a subject has:

- a BKS < 80, there is an approximately 60% chance that they are awake or in N1 stage sleep. Thus, BKS < 80 is a fair estimate of wakefulness.
- a BKS > 110, there is an approximately 80% chance of them being in N2, N3 or REM sleep. Thus, SQ (the percent of the NP with BKS > 110) is an excellent estimate of the quality of sleep and has some relevance to sleep architecture.
- lost the peak of BKS > 110 (see below) then they will have lost 90% of slow wave sleep and 60% of REM sleep.

**Fig. 2:**
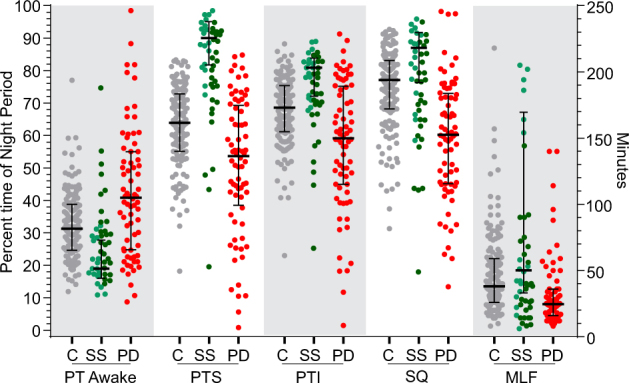
The distribution of PKG sleep parameters in Controls, PwP and during Sleep Studies. This shows the PTA, PTI, PTS and SQ (as percentages, left *Y* axis) and MLF (shown in minutes, right *Y* axis). Controls subjects (C, are shown as grey circles), subjects who had Polysomnography (sleep study—SS, as green circles, with those reported as normal, or normal with caveats, in light green circles) and PwP (PD, as red circles). The bars show median and interquartile range (in the case of subjects having PSG, the bars are for the data from normal subjects)

The five parameters (PTI, PTS, SQ, PTA and MFL) described in the Methods were plotted (Fig. 2) (Table 2). Each parameter has a binomial distribution with a clear central tendency as demonstrated by the small interquartile range around the median, which also approaches the mode (by inspection). Each distribution tends to have a longer 'tail' of ~10–15% of cases trailing into lower percentiles and in considering these it is relevant to recall that some of these controls subjects may have sleep disorders. Inspection of these plots (and Table 2) indicates that in control subjects, median time awake (PTA) was 31%, median sleep efficiency (PTS) was 69%, median sleep quality (SQ) was 77% and median fragment length (MFL) was 38 min (IQR:26–59). As expected PTS was correlated with PTI (*r*^2^ = 0.82), but PTI was slightly larger than PTS (median 3.2%, IQR: 1.4–5.8% of NP).

**Table 2:** The Statistical characteristics of the PKG’s sleep parameters for three participant groups

	Subject	Median	IQR	*p* ^†^
Percent time awake	C	31	14	0.0002
	PD	41	30	
	PSG	19	12	
Percent time sleeping	C	64	18	0.0001
	PD	54	31	
	PSG	90	13	
Percent time immobile	C	69	14	0.0002
	PD	59	30	
	PSG	81	12	
Sleep quality	C	77	15	0.0001
	PD	60	28	
	PSG	87	15	
Median fragment length	C	38	33	0.0001
	PD	25	20	
	PSG	50	137	

^†^ Mann Whitney

### PKG sleep scores and PSG

Forty-six subjects without PD who were investigated with a sleep study (mostly for suspected sleep apnoea) wore a PKG for the evening of the PSG. They were grouped according to the PSG findings as:

Normal (*n* = 10): no abnormality was described in the report and sleep parameters were normal. Median age was 29 y.

Normal minus (*n* = 8): these were described as 'normal sleep study' but mention was made of some aspect such as increased leg movements, oxygen saturation changes or sleep fragmentation. Median age was 42 y.

Abnormal (*n* = 28): these were all cases reported as abnormal even though description ranged from 'mild' abnormality to 'severe'. Median age was 46 y.

The same PKG 'sleep' parameters described in Table 2 were plotted in Fig. 1. The PTA was less, and the other percentages higher for 'Normal' and 'Normal minus' PSG subjects than for the Control subjects (Fig. 1). Although the scores of the 'abnormal' subjects were worse than the other two categories of PSG subjects (dark green circles in Fig. 1), the scores from the normal PSG were better than the older control subjects discussed above. Even the abnormal PSG cases tended to be similar to scores of the older Controls, with the exception of the amount of time sleeping, which was reduced in the normal Controls. This may have been due to the way that sleep efficiency was estimated or simply the poor sleep efficiency of the older person.

**Fig. 3:**
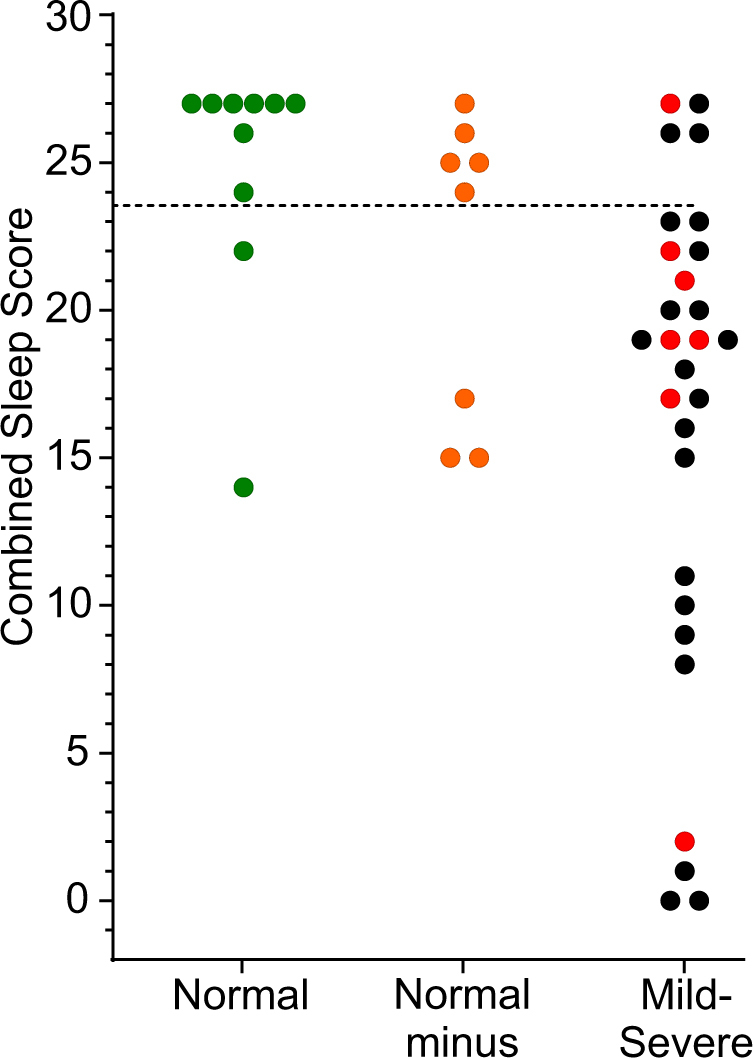
The PKG’s sleep score compared with polysomnography reports. This shows the CSS (*Y* axis) of subjects who underwent a PSG. Subjects are grouped according to whether the sleep study was reported as: 'Normal' (green circles), 'Normal but with caveats' (orange circles) or 'abnormal' (black circles). The red dots refer to subjects in whom the sleep abnormality was reported as 'mild'. The dotted line is where a Receiver–Operator curve provided the best selectivity (86%) and specificity (80%)

None of the five PKG parameters individually provided clear separation between normal and abnormal sleep studies. To permit scores to be added, they were normalised according to their percentile ranking (0–10th percentile, '0'; 10–20th percentile, '1'; etc. to 90–100th percentile, '9'). Various permutations and combinations of adding these five parameters were produced to find the combination that best predicted the PSG diagnosis. The combination that provided the best prediction (Combined Sleep Score—CSS) was the sum of the PTI, PTS and SQ. This was plotted for each subject according to the clinical diagnosis (Fig. 3) and a score of CSS = 23.5 provided a separation between normal sleep subjects and those with an abnormal PSG report with a sensitivity and specificity of 86 and 80%, respectively.

### PKG sleep scores in PD

Figure 2 shows that the distribution, median and interquartile range of the five main parameters (PTI, PTS, SQ, PTA and MFL) for PwP were significantly different from controls (Table 2). The greatest difference between Control subjects and PwP was in Sleep Quality (expressed as both percentages and rank percentiles). This is in keeping with the observation that the peak of BKS ≥ 111 in the night period was often attenuated or absent in PwP (Fig. 1b).

**Fig. 4:**
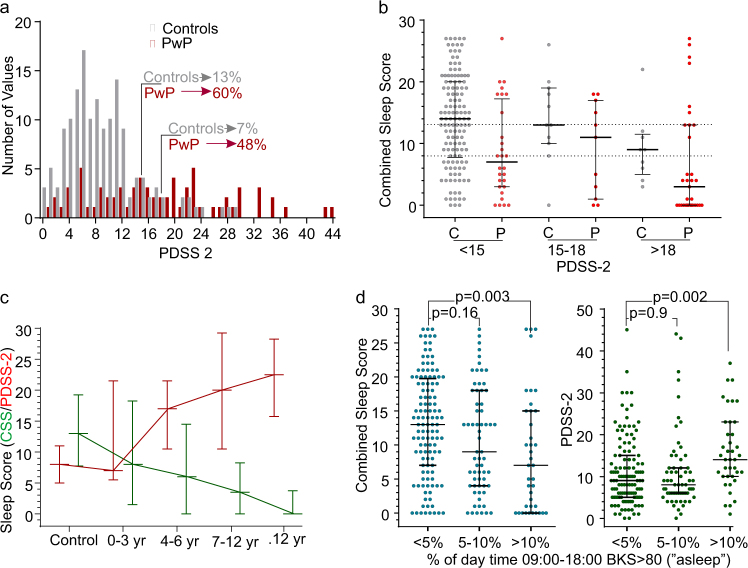
Comparison of PKG score and PDSS 2 in Controls and PwP. **a** This shows cumulative frequency histograms of PDSS 2 scores for Control subjects and PwP. The scores from the two populations are significantly different with between 7 and 13% of Controls having abnormal sleep scores (depending on whether PDSS 2 scores of 15 or 18 are used as the upper limit of normal). **b** This shows the CSS scores of Controls (C) and PwP (P), according to their PDSS 2 scores. The Scores of Controls whose PDSS 2 < 15 are significantly different from PwP with PDSS 2 < 15 (Tukey’s, *p* < 0.05) and Controls whose PDSS 2 > 18 (Tukey’s, *p* < 0.05). The error bars show median and interquartile range, and the dotted horizontal lines represent the median and 25th percentile of the CSS for the whole control population. **c** This shows the PDSS 2 (red lines and bars) and CSS (green line and bars) plotted against duration of disease. The bars show the median and interquartile range. The controls refer to the respective data from Fig. 1. **d** This shows the CSS (left three plots, blue dots) and PDSS 2 (right three plots, green dots) plotted accruing to the amount of time immobile (asleep) in the day

The PDSS 2 in Control subjects and PwP were plotted (Fig. 4a). The upper limit for clinically relevant PD-specific sleep disturbance for the PDSS has been variously quoted as PDSS 2 = 18^19^ and greater than PDSS 2 = 15.^20^ According to the PDSS 2, 13, or 7% of control subjects have clinically relevant sleep disorders (cut-off 15 or 18, respectively), whereas 60 or 52% of PwP have clinically relevant sleep disorders (cut-off 15 or 18, respectively).

The three parameters used for the CSS that best predicted PSG diagnosis was produced for each PwP and control subject (data not shown). CSS for PwP were significantly different from the CSS of controls (Kruskal Wallis/Dunn’s Multiple Comparisons test *p* < 0.0001). The CSS for controls and PwP was then compared with their PDSS 2 scores. There was a technical problem with this comparison: most of the CSS is distributed over the normal range (~90%) whereas only about 30% of PwP had CSS > 25th percentile of controls (in keeping with the prediction of the PDSS 2 that only 30% of PwP have normal sleep). In comparisons, most of the PDSS 2 ranges across abnormal scores with 75% of control subjects having a PDSS 2 < 12 (i.e., normal). This means that the relationship between CSS and PDSS 2 cannot be linear. Nevertheless, the relationship between PDSS 2 and CSS, showed a significant correlation (*p* = 0.0002 Fishers exact test).

Figure 4b shows that most control subjects with a PDSS 2 < 15 had high CSS, whereas those with a PDSS 2 > 18 also had lower CSS. When the PKG’s sub-scores (Fig. 1) were examined for those PwP whose PDDS 2 was <15, it becomes apparent that their PTA is much similar to controls and much less than other PwP. However, PTI and PTS are similar to other PwP suggesting that the reason for their low CSS is fragmentation and early loss of the high BKS that are found with higher stages of sleep. This suggests that even though the duration of sleep may be normal, the quality is impaired. This seems to be the case very early in disease (Fig. 4c).

This was further examined by comparing the PKG sleep sub-scores (Fig. 1) with each of PDSS 2 questions (Table 3). Scores relevant to night time movement (Q1–6 and Q8–12) were examined using the Mutual Information Test, which is an effective measure for quantifying any relationship (linear or nonlinear) between two random variables.^21^ The greatest information about the PDSS 2 came from SQ and PTA, especially for Q1–4 and Q8. Q1–3 relate to a person’s assessment of their sleep quality, Q4 relates to restless legs and Q8 relates to nocturia. It is also relevant that using the mutual information test, it was apparent that each of the PKG sub-scores could be predicted with a high degree of accuracy if one of the other scores were known, suggesting that they may not be independent variables. Taken together this suggests that the PKG’s measure of sleep relates to a subject’s self-assessment of the quality, wakefulness and restlessness.

**Table 3:** The normalised mutual information between each pair of sub-scores in WSS (in percentage form) and PDSS-2

	Q1	Q2	Q3	Q4	Q5	Q6	Q8	Q9	Q10	Q11	Q12
PTA	0.42	0.43	0.44	0.41	0.37	0.32	0.44	0.35	0.35	0.33	0.32
PTI	0.44	0.45	0.46	0.4	0.38	0.34	0.47	0.36	0.35	0.36	0.35
PTS	0.44	0.43	0.47	0.42	0.4	0.36	0.48	0.37	0.37	0.37	0.35
SQ	0.46	0.45	0.48	0.42	0.39	0.36	0.47	0.39	0.39	0.36	0.36
MFL	0.26	0.26	0.3	0.25	0.25	0.23	0.3	0.25	0.25	0.18	0.22

Finally, it is interesting to compare CSS from the single PSG night with the people aged over 60 without PD and PwP. The scores from subjects undergoing a PSG study were substantially higher than those from the older cohort at home. Indeed, the cut-off for a normal PSG study would have excluded about 80% of the control subjects aged over 60.

### Sleep scores and duration of disease

Duration of disease at the time of study was known in 44 PwP. The PDSS 2 scores and the CSS were plotted against the duration of disease (Fig. 4c). Although there was wide variation, there is a very clear trend for both sleep scores to deteriorate as disease progresses. Even by 3 years of disease, 50% of subjects had abnormal PKG sleep scores and many subjects had abnormal PDSS 2 scores early in disease.

### Day time sleep

Previously we showed that the proportion of time with BKS > 80 (PTI) in the day time (between 09:00–18:00) is a measure of day time sleep. Subjects were grouped according to whether their PTI was <5% (median), 5–10% (median to ninetieth percentile) or >20% (associated with low cognition—data not shown) and their CSS and PDSS 2 were plotted (Fig. 4d). More severe daytime sleepiness was associated with worse night time scores, in particular PTI, PTS and SQ.

The PKG measures bradykinesia and dyskinesia over the 6 days of recording. Sleep as measured by PDSS 2 or the PKG was not related to the severity of bradykinesia as measured by the PKG. While the number of cases with dyskinesia was small, there was no significant relationship between the presence of dyskinesia and either sleep measure.

## Discussion

The findings of this study suggest that the PKG’s measures of sleep provide a fairly accurate guide to the duration, quality, wakefulness and fragmentation of sleep. Possibly only the Sleep Quality relates directly to reduction in higher sleep stages, whereas the other scores together indicate whether sleep is 'normal' or 'not normal' without indicating specifically what aspect of sleep is changed. Support for this conclusion include: (i) the link with BKS > 110 and high sleep stages, (ii) changes in PKG sleeps scores linked to Q1–4 of the PDSS 2, (iii) change in scores with progression of disease and prediction of day time sleep and (iv) the relationship with the PDSS 2.

Actigraphy has previously been reported to provide a reasonable measure of sleep.^13^ Typically accelerometry uses a threshold, as was the case in detecting daytime somnolence in a previous study.^16^ However, it is uncommon to examine the distribution of activity to obtain some measure of sleep level, as we have performed here. Empirically, low levels of acceleration over a 2 min period are likely to represent low muscle activity or tone. As the N2, N3 and REM sleep stages are associated with relative muscle atonia, it would be plausible that the proportion of time spent being very immobile (i.e., SQ) would relate to higher stages of sleep. The association between BKS > 110 and higher sleep stages (Fig. 3a) supports this conclusion and future studies combining PSG and PKG studies may show further information about predictions with quality of sleep architecture. It is likely that this would be improved with correlations of heart rate.^22^

Sleep efficiency—the proportion of time in which sleep was attempted that was actually spent in sleep—is also an important measure. In the sleep laboratory setting, 'lights off' and 'lights on' are in the hands of the lab technicians whereas accelerometry has no reliable mechanisms to address this. Although accelerometry can determine when someone is lying recumbent, this does not necessarily mean the person is attempting sleep (an associated light sensor would help clarify this situation). Most people were asleep within 30 min of the start of the assessment period used by the PKG (see Supplementary Figure 1). However, even 30 min beyond this 7-h period would have a significant effect on all the sleep scores. This may account for some of the differences between sleep measures obtained by PSG and those obtained by PKG and it will be difficult to resolve without some direct measure of ambient light.

Markers such as PTI have previously been shown to be a measure of sleep efficiency.^14^ However, microarousals can occur during sleep and may be falsely recognised by accelerometry as a period of wakefulness, thus leading to underestimation of sleep efficiency. To address this, PTS (percent of time sleeping) was proposed. In subjects for whom movements in sleep were relatively infrequent, PTI and PTS would be similar whereas for those with microarousals during their sleep, PTI would substantially be less than PTS. This proved to be the case, and was especially apparent in subjects with sleep disturbance (measured by PDSS 2). Indeed, PwP (and controls) with an abnormal PDSS 2 score had twice the proportion of PTS in the lowest decile than those whose PDSS 2 were normal.

PSG is widely accepted as a 'gold standard' but many people do not sleep well on the first night in a laboratory^23^ even when PSG is performed at home.^8,9^ Nevertheless, it is surprising that the PKG’s Combined Sleep Score that best separated normal sleep from abnormal sleep was so high (23.5): it was ~85th percentile of the CSS of the control subjects aged over 80. These observations should be considered in the light of data of PSG in normal subjects^11^ and in people with PD.^5^ These studies show that total sleep time is reduced and there is a shift in sleep stages toward lighter sleep with age.^11^ This is apparent even in subjects without subjective sleep complaints and does have variation with gender. The findings were similar in PD subjects,^5^ whose sleep time was shorter and sleep efficiency was reduced. However, there was an even greater shift toward lower sleep stages in the PD group than in case matched controls even though arousals or primary sleep were not increased. Taken together this suggests to us that accelerometry is useful in detecting changes that relate to duration and to lower sleep stages, but is less useful in detecting the changes that are related to sleep related breathing disorders. While the fairly high concordance between the PKG scores and the PSG diagnostics provides broad validation, it does indicate that more studies of age matched controls are required and that accelerometry may not be useful in detecting sleep related breathing disorders or periodic limb movements without associated cardiac measures.^22^

The PDSS 2 is a comprehensive questionnaire that asks about night time sleep patterns (questions 1–3), factors that might awaken a subject from sleep (questions 4, 5, 8, 10,11,15), other events that occur in the night (questions 6, 7, 9, 12,13,14).^2^ Because the PDSS-2 scores is weighted to factors that *cause* sleep disturbance rather than the extent of sleep, number of awakenings and quality of sleep, it is unlikely to fully concord with night time accelerometry. On the other hand, there is good correlation with the sub scores that do relate to quality and quantity of night time sleep. A further difficulty is that ~75% of the PKG’s scores relates to sleep patterns of the 'normal' elderly sleeper whereas ~75% of the PDSS 2 relates to factors causing abnormal sleep. The PDSS 2 is also non-linear: the transition from normal to moderate is by an increment of '2' and so also is the transition from moderate to severe. These are not so much criticism as important reasons why concordance can be difficult. Nevertheless, the similarities in detecting abnormal sleep and sleep progression with disease duration and correlation with day time sleepiness suggest that there is inherent agreement between the two ways of measuring nocturnal sleep in PD.

In conclusion, the CSS shows promise as a quantitative score for assessing sleep in Parkinson’s disease. Further study is required to understand its relationship to sleep architecture, although the SQ score shows promise in this respect. Further studies are also required to establish whether the CSS or similar accelerometry scales could be developed as a tool to predict those subjects who do or do not need PSG.

## Methods

### Subjects

Approval for these studies were provided by St Vincent’s Hospital Melbourne Ethics Committee and Monash Health Ethics Committee. All subjects gave consent to participate and for the use of their data. Methods were performed in accordance with relevant regulations and guideline

Control subjects: a PKG logger was worn on each wrist by 155 subjects aged over 60 and without a history or concern of a neurodegenerative disorder. Subjects were not excluded because of the presence of diabetes, cardiac or respiratory problems that might affect sleep and these were assumed to be present to the same extent ad the PD subjects who were of similar age. Only three subjects reported having a diagnosed sleep disorder and the Body Mass Index was the same as the PD subjects. At the time of wearing the logger, they filled out a PDSS 2. Subjects were recruited from Probus clubs, University of Third Age, Bowling Clubs and from the Parkinson’s Day Walk.

PD subjects: 72 people with PD (PwP) attending the Movement Disorder Clinic at St Vincent’s Hospital wore a PKG logger as part of their routine care and were asked to fill out a PDSS 2 questionnaire. Subjects had PD for a median of 6 years IQR:4–11 years and range 1.5–39 years).

PSG subjects: 46 subjects underwent PSG. Most were referred for further investigation of a suspected sleep disorder. Ten had normal studies and their mean age was 29 y (IQR: 26.5–46.5 y). The mean age of the remaining subjects with abnormal studies was 45 y (IQR: 38–53 y).

### The Parkinson’s KinetiGraph system

The PKG (Global Kinetics Corporation) consists of a wrist worn data logger that contains an accelerometer and memory sufficient for 10 days of continuous recording. When recording was completed, data were downloaded and analysed by proprietary algorithms that calculate a BKS and a dyskinesia score (DKS) every 2 min.^24,25^ In this study the PKG logger was worn continuously throughout the day and night for 6 days. PD subjects wore the PKG on the wrist of the most affected side. PSG subjects wore the PKG on the dominant hand overnight for the duration of the PSG study. The control subjects wore the PKG on both wrists for 6 days and data were taken from the wrist with the highest BKS.

### Definitions of PKG activity used in this study

NP: The period between 23:00–06:00 on the days that the PKG was worn.

BKS: BKS is calculated every 2 min throughout the period of time that the logger is worn. The median BKS is the median value of BKS between 09:00–18:00 and correlates with Unified Parkinson’s disease Rating Scale, part III (UPDRS III) for the patient.^24,26^

DKS: DKS is calculated every 2 min throughout the period that the logger is worn. The median DKS is the median value of DKS between 09:00–18:00 and correlates with Abnormal Involuntary Movement Scales (AIMS) for the patient.^24,26^

PTI: This is the proportion of BKS in a period of interest (e.g., the NP), whose score is greater than 80. In previous studies,^16,18^ the PTI in the period 09:00–18:00 was found to correlate well with sleep as measured by ambulatory PSG. As PTI an estimate of the proportion time asleep it is in effect an estimate of sleep efficiency.

PTS: Movement can occur in sleep and so PTI may underestimate sleep disrupted by movements from periodic limb movements or micro-arousals from either obstructive sleep apnoea or those that are spontaneous in nature. To address this, if 4 of 7 consecutive BKS (14 min) were >80 then the central epoch (4th of 7) was defined as 'sleep epoch'. The consecutive BKS were 'slid' forward in time by 1 BKS (i.e., 6/7 of the previous BKS were included and as before, if 4/7 were >80 then the 4th was defined as a Sleep Epoch). This in effect applies a smoothing function to the PTI and is another measure of sleep efficiency that should be less affected by periodic limb movements or arousals.

MFL: The extent to which sleep is fragmented may also reflect the quality of sleep. A fragment of sleep is defined here as a run of BKS > 80 and the length of that fragment is the number of BKS in that sequence. As BKS are 2 mins long, the length, in minutes is twice the number of BKS. In most subjects, the distribution of fragment length is markedly hyperbolic, but even though there is a high proportion of short fragments, most of the sleep (immobility) results from a small number of long fragments. Thus, a measure of sleep fragmentation would be to estimate the MFL in minutes.

SQ: This is the percent the NP with where BKS > 110 and, as elaborated in Results section, has some relation to sleep architecture and is used here asan estimate of the quality of sleep.

PTA: BKS < 80 is used as an estimate of time awake.

NP: In the PSG lab, this is the proportion of time spent asleep (determined from the electroencephalograph) during the period from lights OFF–ON. This is difficult at home with the PKG because it is only possible to assess when sleep began and not the period over which sleep was attempted (i.e., in bed and trying to sleep). Instead the period from 23:00–06:00 was used as the period of sleep: ~75% of people were asleep at 23:00 (or within 30 mins of 23:00) and >90% slept till at least 06:00 (Supplementary Figure 1).

### Polysomnography (PSG)

All subjects (except those having PSG) filled out the PDSS 2.^2^ PSG participants underwent a routine in-lab overnight sleep study using a commercially available PSG system (Grael Sleep System, Compumedics). electroencephalograms, electrooculograms, chin electromyogram (EMG), electrocardiogram, left and right leg EMG and body position were recorded. Oxygen saturation (SpO_2_) was measured by pulse oximetry, thoracic and abdominal breathing movements recorded via uncalibrated respiratory inductance plethysmography, and airflow was recorded via nasal pressure. Sleep, arousals and respiratory events were scored in 30 s epochs according to standard criteria (American Academy of Sleep Medicine Criteria^27^).

### Data availability

All data generated or analysed during this study are included in this published article (and its supplementary information files).

## Electronic supplementary material

Supplementary Figure 1 Caption

Supplementary Figure 1
