# Supplementary material for: The use of accelerometry as a tool to measure disturbed nocturnal sleep in Parkinson’s disease
Source: NPJ Parkinsons Dis. 2018 Jan 10;4:1. doi: 10.1038/s41531-017-0038-9 (PMC5762674; doi:10.1038/s41531-017-0038-9)
Supplement: Supplementary file 1 — Supplementary Figure 1 Caption [file 41531_2017_38_MOESM1_ESM.docx]

## Supplementary Figure 1

## Duration of sleep in Normal and PD subjects

The left Y axis shows the minutes after 23:00 that sleep commenced and the minutes before 06:00 that sleep ended (non-continuous axis): minutes = 0 indicates sleep at 23:00 or awake at 06:00. Green and red circles are Controls and PwP respectively. The right pair of columns shows show the same data, represented as percent of the 7 hours between 23:00 and 06:00 (right Y axis).
