# Supplementary figures and images for: The use of accelerometry as a tool to measure disturbed nocturnal sleep in Parkinson’s disease
Source: NPJ Parkinsons Dis. 2018 Jan 10;4:1. doi: 10.1038/s41531-017-0038-9 (PMC5762674; doi:10.1038/s41531-017-0038-9)

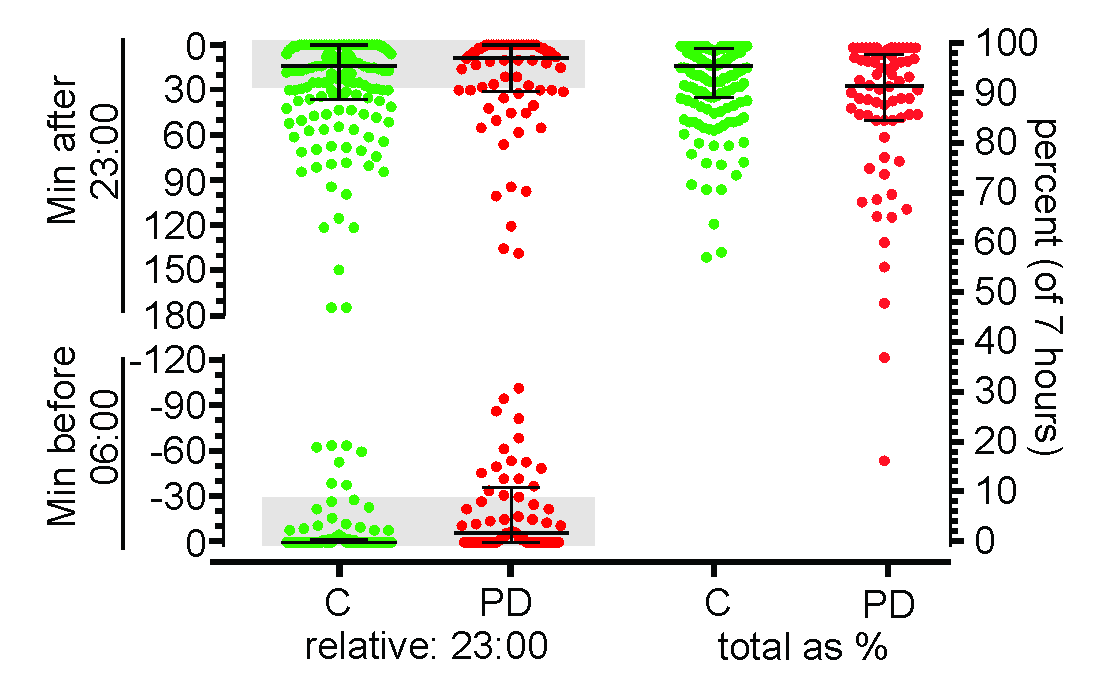

Supplement: Supplementary file 2 — Supplementary Figure 1 [file 41531_2017_38_MOESM2_ESM.tif]
